# Effect of a plant-based hemostatic agent on microleakage of self-etching adhesives

**DOI:** 10.4317/medoral.17959

**Published:** 2012-12-10

**Authors:** Soley Arslan, Hüseyin Ertaş, Yahya O. Zorba

**Affiliations:** 1 DDS, PhD, Instructor, Department of Restorative Dentistry and Endodontics, Faculty of Dentistry, Erciyes University, Kayseri, Turkey; 2 DDS, PhD, Assistant Prof, Department of Endodontics, Faculty of Dentistry, Katip Çelebi University, İzmir, Turkey; 3 DDS, PhD, Assistant Prof, Department of Restorative Dentistry and Endodontics, Faculty of Dentistry, Erciyes University, Kayseri, Turkey

## Abstract

Objective: This in vitro study evaluated the effect of Ankaferd Blood Stopper (ABS) contamination on the microleakage of one-step and two-step self-etching adhesives. Study design: Class V cavities were prepared at the cemento-enamel junction on both buccal and lingual surfaces of 60 freshly extracted human molars. Teeth were randomly assigned into three groups according to contamination material applied (Group I, no contamination; Group II, blood contamination; Group III, ABS contamination). In contaminated groups, one drop of blood and ABS solution was applied directly to the dentin surface and air-dried. Each group was further divided into two subgroups according to bonding agent used [Group A, Clearfil SE Bond (two-step self-etching adhesive); Group B, Adper Easy One (one-step self-etching adhesive)]. Adhesive materials were applied according to the manufacturers’ recommendations. The specimens were restored using a universal microhybrid composite (Arabesk). After thermocycling (5000x, 5°C – 55°C) and immersion in a 0.5% basic fuchsin, dye penetration was evaluated under a stereomicroscope. Statistical analysis was performed with Kruskal-Wallis and Mann-Whitney U tests at p < 0.05. Results: Significantly higher microleakage scores were observed when one-step self-etching adhesive was applied to blood- and ABS-contaminated dentin. However, when a two-step self etching adhesive was used, microleakage was observed only following blood contamination, not following ABS contamination. Conclusions: Although, blood contamination before adhesive application resulted in increased microleakage with both one-step and two-step self-etching adhesive systems, ABS contamination did not affect microleakage when a two-step self-ething adhesive system was used.

** Key words:**Ankaferd Blood Stopper, blood, microleakage, self-etching adhesive.

## Introduction

Adhesion to dentin has been the subject of considerable interest over the last few decades (1). Good bonding to the tooth surface is necessary both for retention and for the prevention of microleakage around the restoration margins (2,3).

Microleakage has been defined by Sidhu and Henderson (4) as the clinically undetectable passage of bacterial fluids, molecules and/or ions between the cavity wall and the restoration material. Marginal microleakage can cause marginal staining, adverse pulpal response, postoperative sensitivity, recurrent caries and clinical failure of restorations. Preventing microleakage is one of the most important aims of adhesive dentistry (5).

Self-etching adhesives have become popular for their reduced technical sensitivity and user friendliness (6). Two-step self-etching systems combine the etchant and primer in one bottle and the adhesive in a separate bottle, whereas one-step systems combine etchant, primer and adhesive in a single solution. Numerous studies have been conducted comparing the two different types of systems (7,8). Burrow et al. (7) found a two-step self-etching adhesive to have significantly higher bond strength to dentin than an ‘all-in-one’ self-etching adhesive, but found no difference in bond strengths to enamel between one- and two-step systems. However, another study also showed all-in-one adhesives to be less reliable than two-step systems when bonding to enamel (8).

Controlling moisture and contamination is a common problem in restorative dentistry, especially when rubber dam isolation is not feasible (2,3). Saliva and blood contamination control is particularly difficult at and below the gingival margin because of the time required for the incremental placement and polymerization of composite (9). Some authors have stated that bond strength may be reduced when adhesive resin is applied to a contaminated surface (10-12).

Clinicians may rely on hemorrhagic agents to avoid blood contamination, particularly with Class V cavities, which occur near or at the gingival margin where blood contamination is an issue. Ankaferd Blood Stopper (ABS) is a medicament produced from natural plant ingredients that have been used for centuries as hemostatic agents in Anatolia. ABS consists of a standardized mixture of *Thymus vulgaris* (dried leaf), *Vitis vinifera* (dried leaf), *Glycyrrhiza glabra* (dried leaf), *Alpina officinarum* (dried leaf) and *Urtica dioica* (dried root), each of which has some effect on the endothelium, blood cells, angiogenesid, cellular proliferation, vascular dynamics and/or cell mediators (13). *T. vulgaris* has been shown to exhibit varying levels of antioxidant activity, which may help prevent in vivo oxidative damage, including the lipid peroxidation associated with atherosclerosis. *V. vinifera* possesses anti-atherosclerotic and antitumor properties and may enhance resistance towards pathogens. *G. glabra* inhibits angiogenesis, decreases vascular endothelial growth factor production and cytokine-induced neovascularization and possesses anti-inflammatory, anti-thrombin, antiplatelet, antioxidant, anti-atherosclerotic and anti-tumor properties. *A. officinarum* has been shown to inhibit nitric oxide production in lipopolysaccharide-activated mouse peritoneal macrophages. *U. dioica* can produce hypotensive responses through a vasorelaxation effect mediated by the release of endothelial nitric oxide and the opening of potassium channels as well as by negative inotropic action (14).

ABS has been shown to be effective in achieving hemostasis following partial liver excitation in an experimental rat model (15). Furthermore, Trakyali et al. (16) showed that ABS may be used clinically to obtain a blood-free tooth surface during the application of brackets on surgically exposed impacted teeth. This study is the first to examine the effects of ABS on the microleakage of self-etching adhesives used with Class V cavities. The null hypothesis was that ABS application has no effect on the microleakage of resin composite applied in conjunction with different self-etching bonding agents.

## Material and Methods

The study was conducted with 60 extracted non-carious human molar teeth stored at 4°C and used within one month of extraction. Standardized Class V cavities (1.5 mm depth, 3 mm mesiodistal, 2 mm occlusogingival) were prepared at the cemento-enamel junction in both buccal and lingual surfaces, with the occlusal margin located in enamel and the gingival margin in dentin. Cavities were prepared using a diamond bur in a high-speed handpiece with water coolant. A template was used to obtain a uniform kidney-shaped outline. Following preparation, all specimens were randomly assigned into three groups according to the contamination material applied, as follows:

Group I (control group): The end of this sentence should be put point 

Group II: Fresh human blood from a female donor was applied with a brush to cavity surfaces and air-dried.

Group III: One drop of ABS solution (ABS patent number 2009-906002, Ankaferd Drug INC®, Istanbul, Turkey) was applied with a brush to conditioned cavity surfaces and air-dried.

Each group was further divided into two subgroups according to bonding agent used, as follows:

Group A: A two-step self-etch bonding system (Clearfil SE Bond, Kuraray®, Tokyo, Japan) was applied according to the manufacturer’s instructions ( Table 1).

**Table 1:**
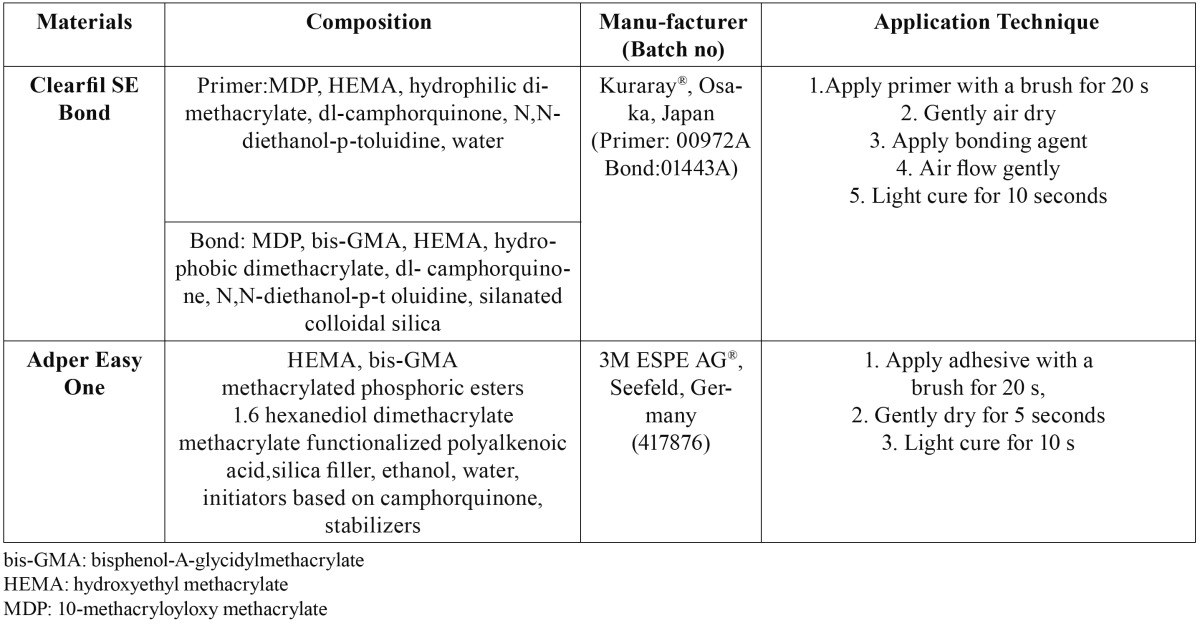
Adhesive Composition and Application Procedures.

Group B: A one-step self-etch bonding system (Adper Easy One, 3M ESPE AG®, Seefeld, Germany) was applied according to the manufacturer’s instructions ( Table 1).

Following adhesive application, cavities were bulk filled with one increment of composite resin (Arabesk, Voco®, Cuxhaven, Germany) and light-cured for 40 s at 600 mW/cm2 using an LED curing unit (Valo, Ultradent Products Inc®, South Jordan, USA) whose output was confirmed using a radiometer (Hilux, Benlioglu®, Ankara, Turkey). Restored teeth were stored in distilled water at 37°C for 24 h and then thermocycled (5°C-55°C, 5.000 cycles, 30 s dwell time). Following thermocycling, teeth were coated with nail varnish up to 1mm from the cavity surface margins, immersed in 0.5% basic fuchsin dye for 24 h and sectioned longitudinally through the center of the restoration using a low-speed diamond saw under water spray. Sectioned restorations were examined under a stereomicroscope (Olympus SZ61, Olympus Optical Co®, Tokyo, Japan) at 25x magnification. Dye penetration along the occlusal and cervical margins of the tooth-restoration interface was evaluated by 2 independent observers and recorded as follows: 0= no dye penetration; 1= enamel penetration; 2= gingival dentin penetration; 3= axial dentin penetration. In cases of disagreement, specimens were reexamined until a consensus was obtained.

Scanning electron microscopic analysis

Two sections of each group (with/without ABS) were flattened using 1000 grit Si-C paper and stored in water solution at room temperature. After 24 hours, the section was gently decalcified (30% hydrochloric acid was applied for 15 seconds, washed and gently air-dried) and 5% sodium hypochlorite was applied onto the surface solution for 120 seconds in order to evaluate the hybrid layer and resin tags formations. After being extensively rinsed with water, the specimens were let dry in air. Then the specimens were gold sputter-coated and finally, the dentin-adhesive interface was observed by SEM. Specimens were sputter-coated with gold and observed with SEM (LEO 440, Oxford, England) at different magnifications ( 3000x - 5000x).

Statistical analysis were performed using the Kruskal-Wallis test. Post-hoc comparisons were done with the Student Newman-Keuls test. Furthermore, the overall microleakage scores were subjected to statistical evaluation using a two independent samples test (Mann-Whitney U).

## Results

Dye penetration scores are given in  Table 2.

**Table 2:**
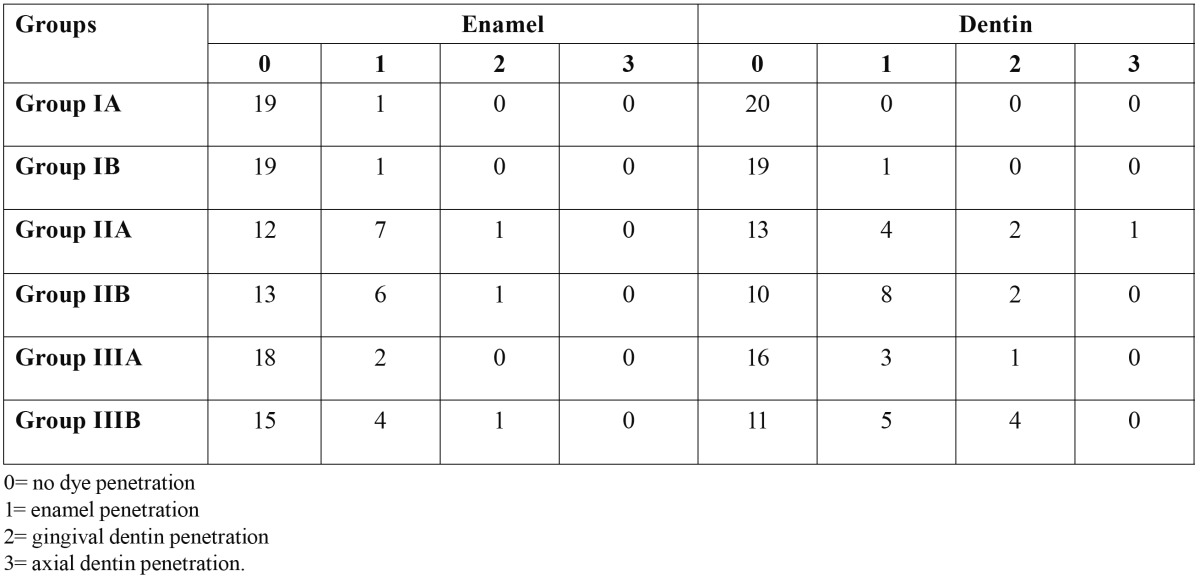
Microleakage Scores.

When the affects of blood contamination were examined, a significant difference was found between the dentin microleakage scores of the control group (IA) and the blood-contaminated group (IIA) when a two-step self-etching adhesive (Clearfil SE Bond) was used (p<0.05), but no difference was found between the enamel microleakage scores of the two groups (p>0.05). When a one-step, self-etching adhesive (Adper Easy One) was used, a significant difference was found between the dentin microleakage scores of the control group (IB) and the blood-contaminated group (IIB) (p<0.05), but no difference was found between the enamel microleakage scores of the two groups (p>0.05)

When the affects of ABS contamination were examined, no significant differences were found in either enamel or dentin microleakage scores of the ABS-contaminated group (IIIA) and the control group (IA) when a two-step self-etching adhesive (Clearfil SE Bond) was used (p>0.05). When one-step, self-etching adhesive (Adper Easy One) was used, a significant difference was found between the dentin microleakage scores of the control group (IB) and the ABS-contaminated group (IIIB) (p<0.05), but no difference was found between the enamel microleakage scores of the two groups (p>0.05).

Comparing enamel and dentin leakage scores within each group, there were a significant differences between enamel and dentin microleakage scores of the ABS-contaminated group when a one-step self-etching adhesive (Adper Easy One) was used (p<0.05), but no difference was found in others groups (p>0.05).

The results revealed that, no significant differences were found in the overall microleakage scores of dentin and enamel, and between the Adper Easy One and Clearfil SE Bond.

**Figure 1:**
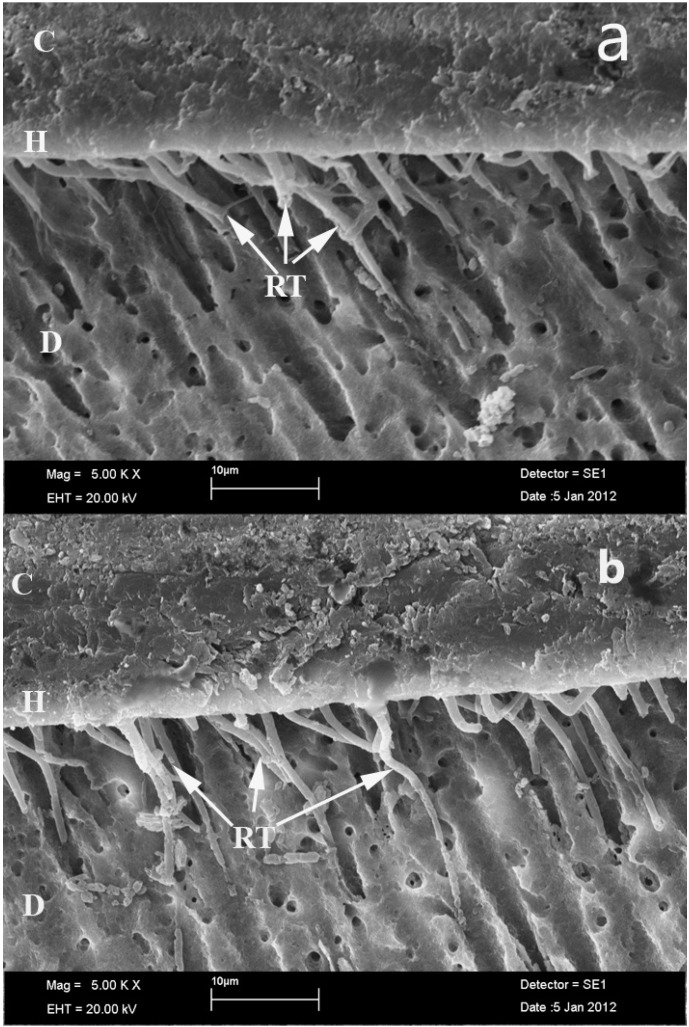
Hybrid layer and resin tags formations of control groups (a.Clearfil SE Bond; b. Adper Easy One) (SEM x5000), C: Composite, H: Hybrid layer, RT: Resin Tag, D: Dentin.

**Figure 2:**
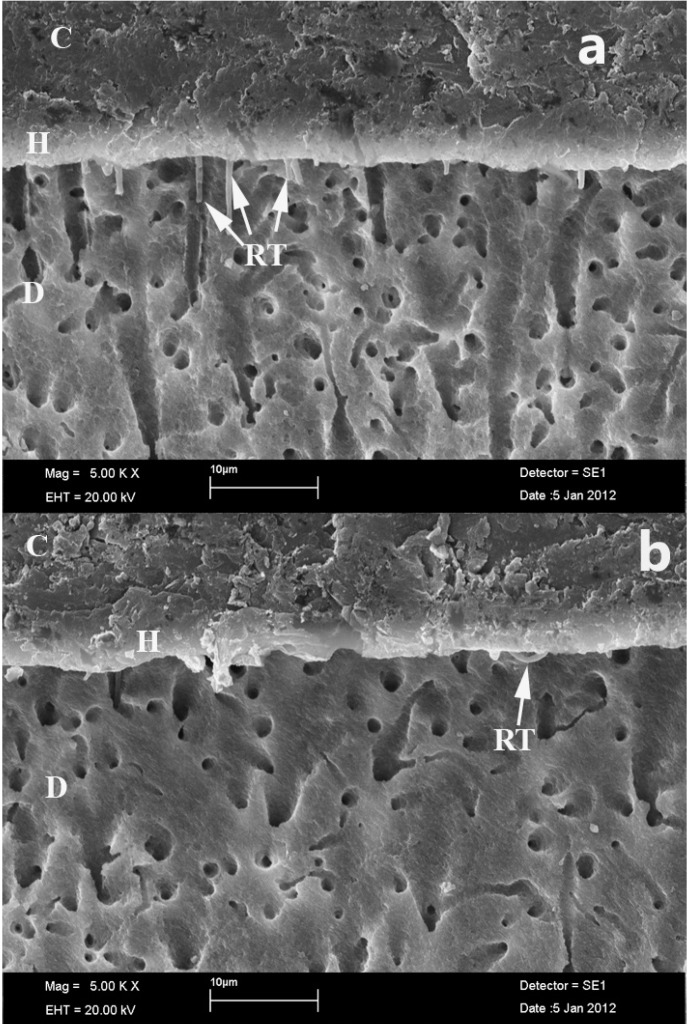
Hybrid layer and resin tags formations of ABS contaminated groups (a. ABS + Clearfil SE Bond; b. ABS + Adper Easy One Bond), (SEM x5000), C: Composite, H: Hybrid layer, RT: Resin Tag, D:Dentin.

SEM images of groups were presented in figure 1,2. In control groups, a continuous and uniform hybrid layer, and resin tags formations were observed at the SEM images of Group IA (Clearfil SE Bond)(Fig. 1), and Group IB (Adper Easy One) (Fig. 1). In ABS contamination groups, continuous hybrid layer and less resin tags formations were observed at Group IIIA (ABS contamination + Clearfil SE Bond)(Fig. 2), and not continuous hybrid layer and less resin tags formations were observed at Group IIIB (ABS contamination+ Adper Easy One)(Fig. 2).

## Discussion

The null hypothesis that ABS application has no effect on the microleakage of resin composite applied in conjunction with different self-etching bonding agents was rejected. In fact, although ABS had no affect on microleakage when a two-step self-etching adhesive was applied, microleakage at the dentin margins increased with ABS contamination when a one-step self-etching adhesive was applied. Blood contamination also resulted in increased microleakage at dentin margins when both one- and two-step self-etching adhesives were applied.

Microleakage is a recognized phenomenon that occurs with time and that may often result in post-operative sensitivity, discoloration and the recurrence of caries in restored teeth. The ability of a composite to minimize the extent of microleakage at the tooth-restoration interface is an important factor in predicting clinical success. Microleakage tests are useful for evaluating the sealing performance of adhesive systems, and microleakage tests combined with thermocycling to accelerate the stresses acting in the oral environment are particularly useful (17).

Previous studies have shown that blood contamination of a prepared tooth can reduce adhesion and retention of restorative materials and thus influence the long-term success of restorations (2,18). The specific effects of blood contamination on microleakage are greatly affected by adhesive system, time of contamination, substrate type and blood type.

This study was conducted using both a one-step and a two-step self-etching adhesive, and contamination occurred before the application of the adhesive system. As a result of fewer components and application steps, self-etching adhesive systems can be applied in less time than other adhesive systems (18,19), thereby reducing the risk of blood contamination in the field of operation (20). The use of a self-etch adhesive avoids the problem of contamination from gingival bleeding as a result of coincidental contact with cavosurface margins that may occur after the rinsing of phosphoric acid gel in cases where cavity preparation is performed without a rubber dam (21). Therefore, this study was conducted using self-etching adhesives.

The finding that blood contamination of dentin resulted in increased microleakage with both one- and two-step self-etching agents is in agreement with previous studies (20,22). Yoo et al. (20) showed that a one-step self-etching adhesive had lower bond strength to contaminated dentin when compared to a non-contaminated control group, and Chang et al. (22) found blood contamination reduced the bond strength of two-step self-etching adhesives to dentin. These results may be attributed to the ability of blood protein to prevent monomers from penetrating enamel and exposed dentine collagen networks (23,24). However, De Calvalho Mendonça et al. (10) showed that blood contamination prior to self-etch primer application did not affect bond strength when a blot-dry technique was used and Oonsombat et al. (25) reported that acidic primer is capable of cleaning blood from the dentin surface.

Ankaferd Blood Stopper is prepared from plant extracts used in Turkish traditional medicine as hemostatic agents. According to Göker et al. (26) ABS appears to initiate the formation of an encapsulated protein network that provides focal points for erythrocyte aggregation, with aggregated blood cells participating to form a mass with the erythrocytes. ABS exposure apparently provides both tissue oxygenation and physiological hemostatis without affecting any individual clotting factor. This unique mechanism of action provides ABS with an advantage over other hemostatically active plants extracts.

ABS can be used to obtain a blood-free, dry enamel surface (16). In the one published study that has reported on the intraoral application of ABS, shear bond strength values of ABS-contaminated group lower than control group (16). In our study, micro-leakage was found to be greater when a one-step self-etching adhesive was applied to ABS-contaminated dentin rather than to normal dentin, non-contaminated dentin. Our findings were in line with previous studies. In these studies, the authors showed that one-step self-etch systems exhibited higher tendency for microleakage when compared with two-step self etch systems (27,28). In addition, in one-step self-etching adhesive applied groups, ABS contamination caused more microleakage to dentin than enamel. This is in line with the fact that enamel is generally considered a more reliable substrate for bonding than dentin (29). It is also accepted that the newer adhesives, dissolved in acetone, alcohol or water solvents, diffuse only into the outer few micron meters of the tissue that has been rendered porous by acid conditioning. Moreover, because self-etching agents has weak acidity, this acid cannot completely remove the ABS layer they are unable to completely remove the ABS layer. Finally, ABS may create a thin, hydrophobic film on the dentin surface that inhibits adhesive penetration into dentin tubules and limits the diffusion of water to the adhesive-composite interface, thereby inhibiting polymerization and consequently weakening the interface (30). The SEM images were verified this findings. In the present study, less resin tags formations occured at SEM images of the Ankaferd applied groups.

In the present study, microleakage increased as a result of blood contamination before adhesive application with both one- and two-step self-etching systems. Microleakage also increased as a result of ABS contamination before adhesive application with the one-step self-etching adhesive, but had no effect on the microleakage of the two-step self-etching adhesive.
